# Sox2 is associated with cancer stem-like properties in colorectal cancer

**DOI:** 10.1038/s41598-018-36251-0

**Published:** 2018-12-05

**Authors:** Koki Takeda, Tsunekazu Mizushima, Yuhki Yokoyama, Haruka Hirose, Xin Wu, Yamin Qian, Katsuya Ikehata, Norikatsu Miyoshi, Hidekazu Takahashi, Naotsugu Haraguchi, Taishi Hata, Chu Matsuda, Yuichiro Doki, Masaki Mori, Hirofumi Yamamoto

**Affiliations:** 1 Department of Surgery, Gastroenterological Surgery, Graduate School of Medicine, Osaka University, Yamadaoka 2-2, Suita, Osaka, 565-0871 Japan; 2 Department of Molecular Pathology, Division of Health Sciences, Graduate School of Medicine, Osaka University, Yamadaoka 1-7, Suita, Osaka, 565-0871 Japan; 3 Department of Medical Physics and Engineering, Division of Health Sciences, Graduate School of Medicine, Osaka University, Yamadaoka 1-7, Suita, Osaka, 565-0871 Japan

## Abstract

Sox2 is known as the undifferentiated cell marker. Recent studies have shown that Sox2 may also be involved in the maintenance of cancer stem cells (CSCs) in skin and bladder cancers. In this study, we aimed to clarify the role of Sox2 in colorectal CSCs. Sox2 expression was measured in colon cancer cells and colorectal clinical samples by qRT-PCR and western blot analysis. To visualize the active Sox2 mRNA production, we generated a Sox2 promoter-dependent DsRed fluorescence emission system. Colon cancer cell lines and colorectal tumor tissues generally expressed the Sox2 protein. Knockdown of Sox2 by siRNA led to increased proliferative activity in Caco2 cells. Kaplan-Meier survival curves showed that the group with high Sox2 mRNA expression had a worse prognosis for relapse-free survival (RFS) than the low expression group (*P* = 0.045, median follow-up 60.0 months). Time-lapse image analysis revealed that most DsRed^+^ cells exhibited typical asymmetric cell division and had higher CSC marker expressions. The DsRed^+^ cells exhibited chemoresistance and they grew slower *in vitro*, yet they established rather larger tumors *in vivo*. Our data suggest that Sox2 may be a potential biomarker for colorectal CSCs.

## Introduction

Colorectal cancer (CRC) is one of the most common cancers and the fourth cause of cancer death in the world^1^. Although surgery and chemotherapy have made great progress in the past decade, 5-year survival remains 50–65% in CRC patients^2,3^. Cancer stem cells (CSCs) play an important role in the recurrence of certain cancers, including colorectal, gastric, and pancreatic cancers^4–7^. The differentiated daughter cells derived from CSCs are sensitive to chemotherapy or radiotherapy, but CSCs are resistant to such treatments and eventually reconstruct the tumor body. Therefore, an ultimate goal of cancer therapy is to develop an efficient strategy to eradicate CSCs.

Sox2, a member of the SRY-related HMG-box (SOX) family, is a transcription factor composed of a transcriptional activation domain and HMG domain with DNA binding ability^8^. Sox2 is involved in the maintenance of an undifferentiated status in the pluripotency of ES cells or pluripotent stem (iPS) cells^9,10^ and plays additional roles in adult tissue homeostasis and regeneration in combination with other synergistic factors^11^. In recent years, aberrant expression of Sox2 has been demonstrated in various types of human cancers, including CRC^12–14^. With regard to CSCs, Sox2-positive skin squamous cell carcinoma cells express high levels of CSC markers CD133 and CD34, and exhibit enhanced tumorigenicity^15^. Zhu *et al*. demonstrated that Sox2-expressing bladder cancer cells express high levels of CSC markers keratin14 (KRT14) and CD44v6, and that ablation of Sox2-expressing cells leads to tumor regression^16^. These findings suggest that Sox2 may be one of the key molecules driving CSCs in skin and bladder carcinomas^15,16^. However, few studies have demonstrated a specific role of Sox2 in colorectal CSCs^17,18^.

In the present study, we show that colon cancer cell lines and clinical CRC samples frequently express basal levels of the Sox2 protein. Because CSCs are thought to exist as a minimal population relative to the whole tumor, we postulated that the colon cancer cells actively producing Sox2 mRNA retain CSC-like properties, such as resistance to chemotherapy and asymmetric cell division^19^. To monitor these cells, we constructed a fluorescence cell visualization system in response to Sox2 promoter activation and tried to elucidate whether Sox2 may drive CSCs in colon cancer.

## Results

### Sox2 gene expression in colon cancer cells and CRC tissues

**Figure 1:**
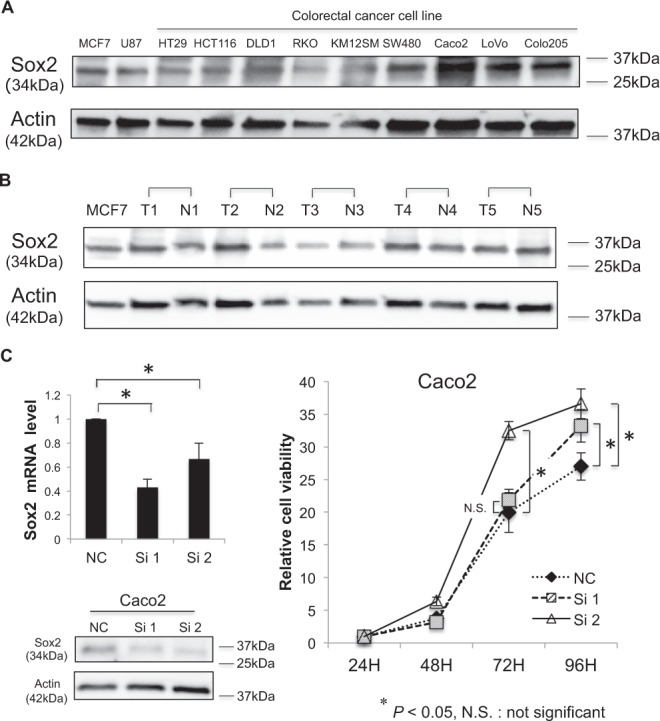
Sox2 was expressed in colon cancer cells and colorectal cancer (CRC) tissues and knocking down Sox2 promoted cell proliferation. (**A**) The protein extract from human colon cancer cells was immunoblotted for Sox2 (top) and actin (bottom). The protein extract from MCF7 (human breast cancer) and U87 (human glioblastoma) cells was used as a positive control. Actin was used as the loading control. Caco2 expressed relatively high levels of Sox2 protein. (**B**) The protein extract from human colorectal tumor (T) or normal colorectal mucosa (N) obtained from clinical CRC samples was immunoblotted for Sox2 (top) and actin (bottom). Actin was used as the loading control. (**C**) Top left: mRNA was extracted from Caco2 cells transfected with siRNAs (50 nM) against Sox2 (Si 1 or Si 2) or negative control (NC) siRNA (50 nM). Values are expressed as the fold value of cells treated with NC. *P < *0*.*05*. Lower left: The protein extract from Caco2 cells transfected with Si 1, Si 2, or NC (50 nM) was immunoblotted for Sox2 (top) or actin (bottom). Actin was used as the loading control. Right: Cell proliferation assay using Caco2 cells transfected with Si 1, Si 2, or NC (50 nM). Values are expressed as the fold value of the number of cells 24 hours after seeding. *P < *0*.*05*.

In western blot analyses, colon cancer cell lines generally expressed the Sox2 protein at levels comparable to positive control MCF7 and U87 cells (Fig. 1A). Clinical CRC samples also expressed Sox2 at moderate levels (Fig. 1B). To explore the potential function of Sox2 in the proliferative activity of colon cancer cells, we treated Caco2 cells that retain relatively high Sox2 protein expression with small interfering RNA (siRNA) *in vitro*. Sox2 mRNA expression decreased after transfection of the two siRNA sequences to 43% and 67% of the negative control treatment and confirmed reduced Sox2 protein expression (Fig. 1C). Cell proliferation assays indicated that siRNA treatment significantly enhanced cell proliferation 96 hours after transfection (Fig. 1C).

### Disease recurrence and Sox2 expression in CRC tissue samples

**Figure 2:**
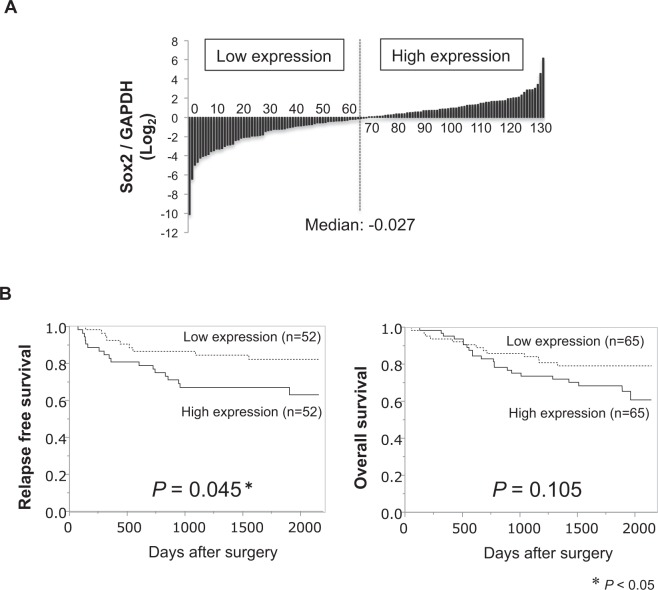
Prognostic evaluation of Sox2 mRNA expression in patients with colorectal cancer (CRC). (**A**) The ratio of Sox2 and GAPDH in CRC tissue samples was examined by qRT-PCR and 130 CRC patients (Stage I/II/III/IV: 17/38/51/24) divided into two groups according to the median value of Sox2 mRNA expression. (**B**) Kaplan-Meier survival curve showing that the high expression group had a worse prognosis for relapse-free survival (RFS) than the low expression group (*P* = 0.045, median follow-up 60.0 months) (left) and a worse tendency for overall survival (OS) than the low expression group (*P* = 0.105, median follow-up 60.7 months) (right).

Sox2 mRNA expression was examined by qRT-PCR and CRC tissue samples from 130 CRC patients (Stage I/II/III/IV: 17/38/51/24), which were divided into two groups according to the median value (Fig. 2A). No significant difference was found in patient background between the high Sox2 expression group (n = 65) and low Sox2 expression group (n = 65) (Supplementary Table 1). Kaplan-Meier survival curves showed that the high expression group had worse prognosis for relapse-free survival (RFS) compared to the low expression group (*P* = 0.045, median follow-up 60.0 months; Fig. 2B). There was also a tendency for worse overall survival (OS) in the high expression group than the low expression group (*P* = 0.105, median follow-up 60.7 months; Fig. 2B). Univariate analysis revealed that tumor depth (*P* = 0.0003), lymph node metastasis (*P* = 0.001), venous invasion (*P* = 0.026), and Sox2 expression (*P* = 0.023) were significant prognostic factors. Multivariate analysis revealed that Sox2 gene expression was an independent prognostic factor for RFS (Table 1, HR 2.529, 95% CI 1.148–5.973, *P* = 0.021), as well as T4 invasion (HR 3.466, 95% CI 1.467–9.192, *P* = 0.004).

**Table 1:** Univariate and multivariate analysis of relapse free survival in 104 colorectal cancers.

Clinicopathological factors	Univariate			Multivariate		
	HR	95%CI	*P* value	HR	95%CI	*P* value
Gender (male/female)	1.093	0.512–2.382	0.818			
Location (rectum/colon)	1.694	0.792–3.652	0.172			
Lymph node dissection (<12/≥12)	0.577	0.137–1.652	0.336			
Depth (T4/others)	4.383	1.938–11.181	0.0003*	3.466	1.467–9.192	0.004*
Lymph node metastasis (positive/negative)	3.774	1.669–9.629	0.001*	2.125	0.894–5.647	0.090
Histological type (por, sig, muc/tub1, tub2)	2.359	0.379–7.950	0.300			
Lymphatic duct invasion (positive/negative)	2.384	0.976–7.127	0.057			
Venus invasion (positive/negative)	2.505	1.108–6.388	0.026*	1.693	0.724–4.432	0.232
Sox2 expression (high/low)	2.453	1.130–5.729	0.023*	2.529	1.148–5.973	0.021*

Abbreviations: por, poorly differentiated adenocarcinoma; sig, signet ring cell carcinoma; muc, mucinous carcinoma; tub1, well-differentiated adenocarcinoma; tub2, moderately differentiated adenocarcinoma;T4, tumor directly invades other organs or structures and/or perforates visceral peritoneum; *P value < 0.05.

### Visualization of cells producing Sox2 mRNA

**Figure 3:**
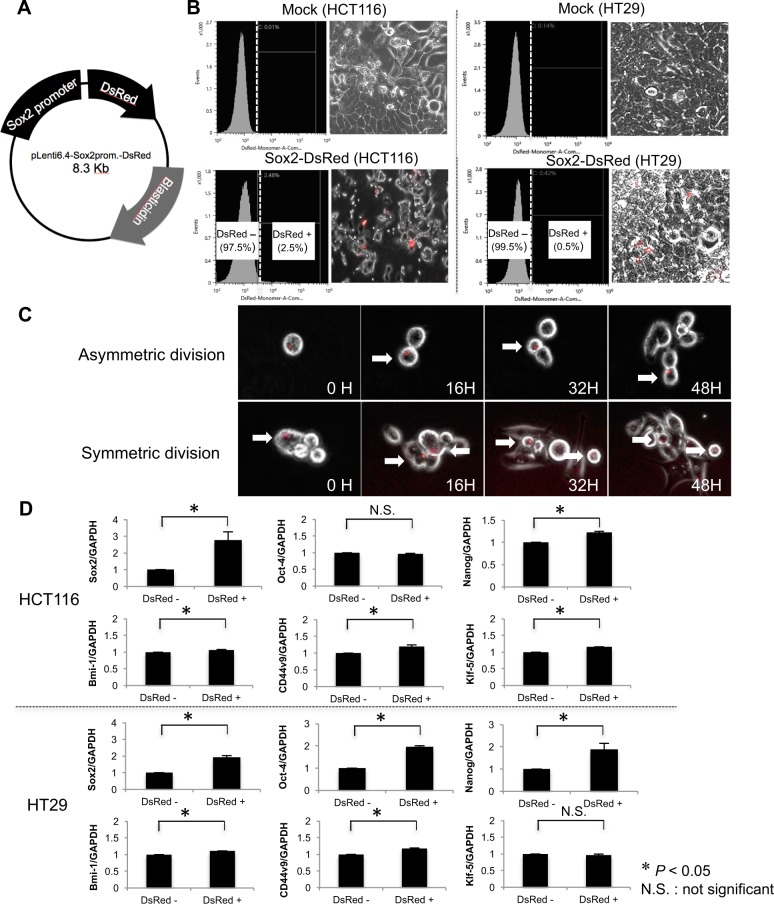
DsRed^+^ (Sox2^+^) colon cancer cells had cancer stem cell-like properties. (**A**) Schematic representation of the plasmid of Sox2 promoter activity-dependent cell visualization system with DsRed fluorescence. (**B**) HCT116 (left) or HT29 (right) cells were infected with empty-vector (top, Mock) or the vector encoding Sox2 promoter followed by DsRed gene (bottom, Infection). The percentage of DsRed^+^ cells was measured by flow cytometry. DsRed^+^ cells were visualized by microscopy as red signal. (**C**) Time-lapse image of DsRed^+^ cells showing asymmetric division of DsRed^+^ cells. Arrows indicate DsRed^+^ cells. (**D**) The expression of undifferentiated markers Sox2, Oct-4, and Nanog, and cancer stem cell markers Bmi-1, CD44v9, and Klf-5 was examined between DsRed^+^ and DsRed^−^ HCT116 (top) or HT29 (bottom) cells. Values are expressed as the fold value of DsRed^−^. Sox2-expressing DsRed^+^ cells often exhibited high expression of undifferentiated markers or cancer stem cell markers. GAPDH was used as the loading control. *P < *0*.*05*.

**Figure 4:**
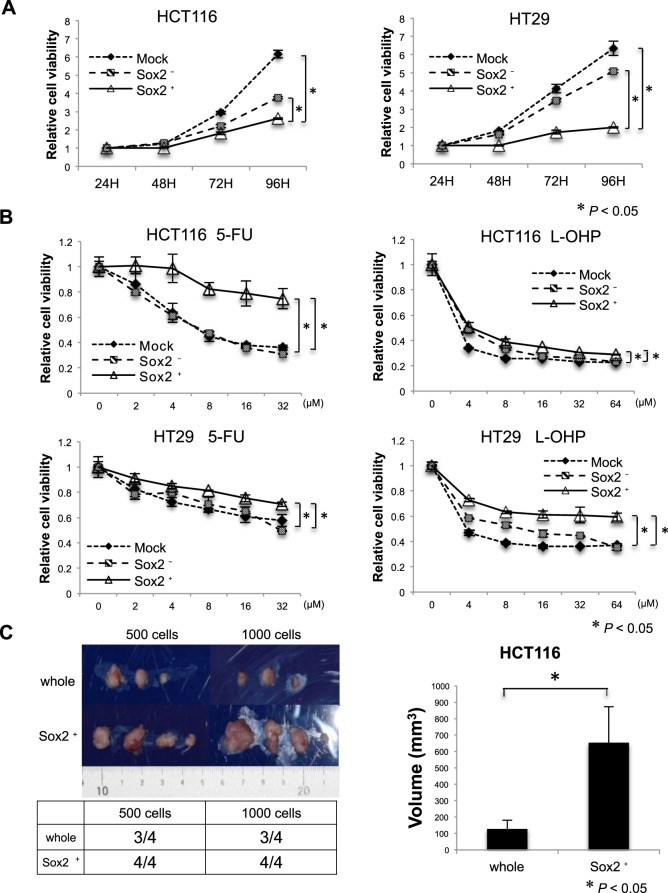
DsRed^+^ (Sox2 active) cells had low proliferation ability, enhanced chemoresistance, and established larger tumors *in vivo*. (**A**) Cell proliferation assay using HCT116 (left) or HT29 (right) cells. Proliferation of Sox2^+^ cells significantly decreased compared to Sox2^−^ cells or empty vector-transfected cells. Values are expressed as the fold value of the number of cells 24 hours after seeding. *P < *0*.*05*. (**B**) The chemoresistance of Sox2^+^ cells to 5-FU or oxaliplatin (L-OHP) in HCT116 (top) and HT29 (bottom) cells. Chemoresistance significantly increased in Sox2^+^ cells compared to Sox2^−^ cells or empty vector-transfected cells. Values are expressed as the fold value of the untreated cell number. *P < *0*.*05*. (**C**) *In vivo* tumor formation. Sox2^+^ or the whole HCT116 cells were subcutaneously injected into the right or left back of mice (n = 4) using 500 or 1000 cells. The frequency of tumor generation (lower left) and tumor volume (tight) was measured. Sox2^+^ cells established larger tumors compared to the whole cells. *P < 0.05.

We constructed a lentivirus vector for Sox2 promoter activity-dependent cell visualization. The DsRed gene was inserted downstream of the Sox2 promoter (Fig. 3A). Flow cytometric analysis showed that the proportion of DsRed^+^ cells among HCT116 and HT29 cells was only 2.5% and 0.5%, respectively (Fig. 3B). After sorting the fluorescent cells, time-lapse imaging revealed that most DsRed^+^ cells (approximately 90%) demonstrated the typical asymmetric cell division that is a hallmark of CSCs^19^ and the daughter cells repeat fast cell division. In contrast, the remaining 10% DsRed^+^ cells underwent symmetric cell division (Fig. 3C). qRT-PCR showed that DsRed^+^ cells had significantly higher Sox2 mRNA expression than DsRed^−^ HCT116 and HT29 cells (Fig. 3D, *P* < 0.05), indicating that DsRed^+^ cells actively produce Sox2 mRNA (hereafter also designated Sox2^+^ cells). DsRed^+^ cells often express high levels of undifferentiated markers Oct-4 and Nanog^20,21^, or CSC markers Bmi-1, CD44v9, and Klf-5^22–24^. Statistical significance (*P* < 0.05) was found for most molecules, except Oct-4 in HCT116 cells and Klf-5 in HT29 cells (Fig. 3D). Compared to empty vector-transfected cells (Mock control) or Sox2^−^ cells, the proliferative ability of Sox2^+^ cells was significantly decreased (*P* < 0.05, Fig. 4A). We also found that both Sox2^+^ HCT116 and HT29 cells had chemoresistance for 5-FU and oxaliplatin (L-OHP) (*P* < 0.05, Fig. 4B). Finally, *in vivo* tumor formation assays indicated that HCT116 cells as a whole had potent tumorigenicity by injecting 500 or 1000 cells (Fig. 4C, Incidence: 3/4 (75%)). On the other hand, Sox2^+^ cells exclusively produced tumors at all sites (Incidence 4/4 (100%)). Moreover, the Sox2^+^ cells produced much larger tumors than whole HCT116 cells (*P* < 0.05).

## Discussion

In this study, we found that colon cancer cells express considerable levels of the Sox2 protein, comparable to the MCF7 and U87 cells used as positive controls. Colorectal cancer tissue samples also expressed the Sox2 protein at moderate levels. In an analysis of clinical CRC samples, high Sox2 mRNA expression was associated with poor prognosis, and multivariate analysis indicated that high Sox2 mRNA expression was an independent prognostic factor for RFS. These results were consistent with previous immunohistochemistry studies of Sox2 in rectal cancer and right-sided colon cancer^18,25^. Knockdown experiments have also shown that the Sox2 transcription factor is involved in cell migration, invasion, and metastasis via activation of the epithelial and mesenchymal transition (EMT) or WNT signaling pathway^12^. These findings suggest that colon cancer cells and CRC tissue samples express the Sox2 protein to some extent, and that Sox2 may play a role in tumor progression and disease recurrence in CRC.

Recent studies have shown that Sox2, a putative undifferentiated marker, may be involved in the behavior of CSCs in skin squamous cell carcinoma and bladder cancer cells^15,16^. On the other hand, few studies have examined the role of Sox2 in colorectal CSCs^17,18^. CSCs have the ability for self-renewal, multi-lineage differentiation, enhanced chemoresistance, and tumorigenicity, and are responsible for tumor maintenance and disease recurrence^26,27^.

We showed increased proliferation of Caco2 cells in knockdown experiments using Sox2 siRNA, indicating that Sox2 may suppress cell proliferation. This result is consistent with the stable overexpression of Sox2 leading to growth inhibition of colon cancer cells^17^. These findings suggest that Sox2 may be associated with quiescent features of CSCs^28,29^. Lundberg *et al*. also demonstrated a spheroid-like growth pattern and high CD44 and CD24 mRNA expression in tumor cells over-expressing Sox2, but CD133 expression was decreased^17^. If Sox2 is really involved in CSC regulation, it could be one reason for the poor prognosis of patients with Sox2-expressing CRC. Notably, Saigusa *et al*. showed that rectal cancer patients who receive preoperative chemoradiotherapy are likely to develop tumor recurrence if the tumors express high levels of Sox2 mRNA^18^, which suggests resistance to treatment with radiation and chemotherapy via a Sox2-mediated mechanism.

Most colon cancer cells may fundamentally retain Sox2 protein expression; however, real CSCs are thought to exist in a small population. This discrepancy between the tissue samples and cell system occurs also with other putative CSC markers such as lgr5 and Dclk1. CSCs are supposed to represent a small population, but these CSC markers are often expressed abundantly in human colon cancer tissues by immunohistochemistry^30,31^. Moreover, a recent study using *in vivo* lineage tracing system showed that lgr5-expressing CSCs expanded within the tumor^32^. Therefore, it is probable that CSCs may expand its population in the heterogenous tumor tissue. In a separate study, we found that the tumor tissue, which was generated via a single cell inoculation of a CSC model cell into mice expressed considerably high Sox2 mRNA (Our unpublished observation, HY and KT). This is not surprising because pluripotent factor Sox2 may help to build up a tissue organ from a single CSC. Considering its nature of pluripotency, it is probable that Sox2 might be secondarily induced when CSCs produce the cancer tissue structures.

To highlight candidate CSCs in which Sox2 transcription is actively driven, we set-up a Sox2 promoter activity-dependent visualization system. We could collect Sox2^+^ living tumor cells by FACS sorting and examine several features specific to CSCs, including chemoresistance, asymmetric division, and *in vivo* tumor growth with relatively few tumor cells.

As expected, the population of DsRed^+^ cells was scant, and these cells retained high levels of Sox2 mRNA, as well as other undifferentiated markers (i.e. Oct-4 and Nanog)^20,21^. The DsRed^+^ cells also expressed high levels of stem cell markers Bmi1, CD44v9, and Klf-5^22–24^. These data suggest that DsRed^+^ cells reflect the production of Sox2 mRNA and may have CSC-like properties. Furthermore, we found that the DsRed^+^ (Sox2^+^) cells acquired chemoresistance to 5-FU and oxaliplatin, and that Sox2^+^ cells exhibited slower growth activity *in vitro*, though they exhibited larger tumor formation *in vivo*. This discrepancy between the *in vitro* and *in vivo* results could be attributed to CSC characteristics. CSCs undergo cell division more slowly than differentiated cells, and the slow-cycling phenotype plays a role in tumor recurrence^28,29^. Sox2^+^ cells are postulated to have the ability to survive and divide in the mouse body, followed by expansive and rapid growth of the differentiated daughter cells, resulting in the establishment of larger tumors. We observed that the DsRed^+^ cells often demonstrated asymmetric division, which is a solid hallmark of CSCs^19^. Notably, the daughter cells generally divided much faster than the DsRed^+^ cells in the time-lapse image analysis. Taken together, these findings strongly suggest that the Sox2^+^ colon cancer cells behave like CSCs.

In this study we focused on the epithelial tumor cells alone. Considering the role of Sox2 in pluripotency, it is probable that Sox2 may exert a role in stromal cells when tumor cells produce the tissue organ involving surrounding cancer stroma. Indeed, we observe that Sox2 is expressed mainly at the epithelial cells but it is occasionally expressed also in stromal cells in tumor lesions of Sox2 promoter responsive GFP mice (our unpublished observation, HY and KT).

In conclusion, our data suggest that Sox2 plays a crucial role in colorectal CSCs. Further investigations using Sox2 transgenic mice are currently underway in our laboratory, and a detailed mechanism of the involvement of Sox2 in CSC production and maintenance is expected to be clarified.

## Materials and Methods

### Cell culture

Human colorectal cancer cell lines HT29, HCT116, DLD1, RKO, SW480, LoVo, Colo205, and Caco2; human primary glioblastoma cell line U87; and human breast cancer cell line MCF7 were purchased from the American Type Culture Collection (Rockville, MD, USA). KM12SM^33^ was a kind gift from Prof. T Minamoto (Cancer Research Institute, Kanazawa University, Japan). Cells were cultured in Dulbecco’s modified Eagle medium (DMEM; Sigma-Aldrich, St. Louis, MO, USA) with 10% fetal bovine serum (FBS) in a humidified incubator with a 5% CO_2_ atmosphere at 37 °C.

### Clinical tissue samples

Tumor tissue and adjacent normal tissues were collected from CRC patients who underwent surgery between 2003 and 2015 at Osaka University Hospital. These samples were immediately frozen in RNAlater (Ambion, Austin, TX, USA) and stored at −80 °C until RNA extraction. This study was approved by the Ethics Board of Osaka University Hospital. All patients provided written informed consent.

### Quantitative reverse transcription polymerase chain reaction (qRT-PCR)

Total RNA was isolated from CRC cells using TRIzol Reagent (Thermo Fisher Scientific, Waltham, MA, USA) and clinical samples using the miRNeasy Mini Kit (Qiagen, Hilden, Germany). The RNA quality was assessed using a NanoDrop ND-2000 spectrophotometer (NanoDrop Technologies, Rockland, DE, USA). Total RNAs were reverse transcribed using the Reverse Transcription System (Promega Corporation, Madison, WI, USA). qRT-PCR was performed with a LightCycler 480 Real-Time PCR system (Roche Diagnostics, Mannheim, Germany) using specific primers (Supplementary Table 2) and a LightCycler-DNA Master SYBR Green I (Roche Diagnostics) or ABI Prism 7900HT Sequence Detection System (Applied Biosystems, Foster City, CA, USA). Relative expression was quantified using the ΔΔCt method^34^.

### Western blot analysis

Cells or tissue samples were lysed in RIPA buffer with protease inhibitors. After centrifuging at 12,000 rpm for 15 min, the supernatant was collected. The protein lysates (30 μg) were separated by sodium dodecyl sulfate-polyacrylamide gel electrophoresis (SDS-PAGE) and transferred to a polyvinylidene difluoride (PVDF) membrane. After blocking, the membrane was incubated with anti-human polyclonal antibodies against Sox2 (Abcam, Cambridge, UK) and actin (Sigma-Aldrich, St. Louis, MO, USA), followed by incubation with the secondary antibody and visualization using the ECL Detection System (GE Healthcare, Little Chalfont, UK).

### siRNA transfection

Sox2 siRNA and negative control siRNA were purchased from Applied Biosystems. Transfection was performed at a final concentration of 50 nmol/L 24 hours after seeding with Lipofectamine RNAiMAX Reagent (Thermo Fisher Scientific).

### Lentivirus vector transfection

The Sox2 promoter followed by the DsRed gene was subcloned into the pLenti6.4/R4R2/V5-DEST (Thermo Fisher Scientific) using LR clonase II (Thermo Fisher Scientific) and the following primers: Sox2 F-primer: 5′-gttgtctattaacttgttca-3′, Sox2 R-primer: 5′-gctgtttttctggttgccgc-3′, DsRed F-primer: 5′-caccatggtggcctcctccgagga-3′, DsRed R-primer: 5′-ctacaggaacaggtggtggc-3′. The promoter region was cloned using GoTaq® Colorless Master Mix. Amplification conditions included 25 cycles of denaturation for 10 s at 98 °C, annealing at 55 °C for 30 s, and extension at 72 °C for 1 min. The PCR product was collected using the QIAquick Gel Extraction Kit. The pENTR 5′-Promoter vector was prepared using Top10 *E*. *coli* and TA cloning. The recombination reaction of pENTR 5′-Promoter vector and pENTR-DsRed vector was performed using LR Clonase II in order to prepare a lentiviral vector. The vector was transfected into 293FT cells with packaging plasmids using Lipofectamine 2000 (Thermo Fisher Scientific) according to the manufacturer’s protocol. Forty-eight hours after transfection, the supernatant was filtered and used for virus transduction into HCT116 and HT29 cells with 5 µM polybrene (Sigma-Aldrich). Stable clones were obtained after selection by blasticidin.

### Flow cytometry

After centrifuging the cells, cell were dissolved in 10% bovine serum albumin/phosphate - buffered saline. DsRed fluorescence was examined by FACS, and the top 5% cells was collected as DsRed^+^ and the bottom 5% cells was collected as DsRed^−^. Fluorescence data were analyzed using a Cell Analyzer SH800 (Sony Corp., Tokyo, Japan).

### Cell viability assay

Cells were seeded at a density of 3–5 × 10^3^ cells per well in 96-well plates. Cell proliferation was assessed using Cell Counting Kit-8 (Dojindo, Tokyo, Japan). For chemosensitivity tests, 100 μl of DMEM containing 5-FU (Kyowa, Tokyo, Japan) or L-OHP (Yakult, Tokyo, Japan) was added 24 hours after cell seeding and cell viability determined 72 hours later.

### *In vivo* tumor formation

Whole HCT116 cells and DsRed^+^ cells were subcutaneously injected into the right and left backs of SCID Beige mice (n = 4 for 500 cells, n = 4 for 1000 cells) in 200 μl DMEM/Matrigel (BD) (1:1). After 4 weeks, the mice were sacrificed.

### Time-lapse imaging analysis

DsRed^+^ cells were separated by flow cytometry and plated at a density of 10^4^ cells in 35-mm dishes in DMEM supplemented with 10% FBS. Image analysis was performed using a BZ-X fluorescence microscope (KEYENCE, Osaka, Japan).

### Statistical analysis

All data are indicated by mean ± standard deviation. Statistical differences were analyzed by the Student’s *t* test for continuous variables and the chi-squared test for the others. Survival curves were drawn using the Kaplan-Meier method and compared using the log-rank test. The Cox proportional hazard regression model was used to estimate the hazard ratio (HR) and 95% confidence interval (CI). All statistical analyses were performed with JMP ver. 13.0.0 (SAS Institute, Inc., Cary, NC, USA). *P* values < 0.05 were considered significant.

### Statement in the Methods

This study was approved by the Ethics Board of Osaka University Hospital (No.15218-4). The animal experiments in this study were approved by Institutional Animal Experiments of Osaka University (No. 27-085-009). All methods were performed in accordance with the relevant guidelines and regulations.

## Electronic supplementary material

Supplementary Figure

## References

[1] Torre LA (2015). Global cancer statistics, 2012. CA Cancer J Clin.

[2] Brenner H, Kloor M, Pox CP (2014). Colorectal cancer. Lancet.

[3] Colvin HS (2014). Cancer stem cells of the digestive system. Jpn J Clin Oncol..

[4] Zeuner A, Todaro M, Stassi G, De Maria R (2014). Colorectal cancer stem cells: from the crypt to the clinic. Cell Stem Cell..

[5] Ishimoto T, Sawayama H, Sugihara H, Baba H (2014). Interaction between gastric cancer stem cells and the tumor microenvironment. J Gastroenterol..

[6] Fitzgerald TL, McCubrey JA (2014). Pancreatic cancer stem cells: association with cell surface markers, prognosis, resistance, metastasis and treatment. Adv Biol Regul..

[7] Luo M (2015). Breast cancer stem cells: current advances and clinical implications. Methods Mol Biol..

[8] Kamachi Y, Uchikawa M, Kondoh H (2000). Pairing SOX off: with partners in the regulation of embryonic development. Trends Genet..

[9] Avilion AA, Nicolisl SK, Pevny LH, Perez L (2003). Nigel Vivian, and Robin Lovell-Badge. Multipotent cell lineages in early mouse development depend on SOX2 function. Genes Dev.

[10] Takahashi K, Yamanaka S (2006). Induction of pluripotent stem cells from mouse embryonic and adult fibroblast cultures by defined factors. Cell.

[11] Sarkar A, Hochedlinger K (2013). The sox family of transcription factors: versatile regulators of stem and progenitor cell fate. Cell Stem Cell..

[12] Han X (2012). Silencing SOX2 induced mesenchymal-epithelial transition and its expression predicts liver and lymph node metastasis of CRC patients. PLoS One.

[13] Li XL (2004). Expression of the SRY-related HMG box protein SOX2 in human gastric carcinoma. Int J Oncol..

[14] Amini S, Fathi F, Mobalegi J, Sofimajidpour H, Ghadimi T (2014). The expressions of stem cell markers: Oct4, Nanog, Sox2, nucleostemin, Bmi, Zfx, Tcl1, Tbx3, Dppa4, and Esrrb in bladder, colon, and prostate cancer, and certain cancer cell lines. Anat Cell Biol..

[15] Boumahdi S (2014). SOX2 controls tumour initiation and cancer stem-cell functions in squamous-cell carcinoma. Nature.

[16] Zhu F (2017). SOX2 Is a Marker for Stem-like Tumor Cells in Bladder Cancer. Stem Cell Reports..

[17] Lundberg IV (2016). SOX2 expression is associated with a cancer stem cell state and down-regulation of CDX2 in colorectal cancer. BMC Cancer..

[18] Saigusa S (2009). Correlation of CD133, OCT4, and SOX2 in rectal cancer and their association with distant recurrence after chemoradiotherapy. Ann Surg Oncol..

[19] Caussinus E, Hirth F (2007). Asymmetric stem cell division in development and cancer. Prog Mol Subcell Biol..

[20] Wen K (2013). Oct-4 is required for an antiapoptotic behavior of chemoresistant colorectal cancer cells enriched for cancer stem cells: effects associated with STAT3/Survivin. Cancer Lett..

[21] Ibrahim EE (2012). Embryonic NANOG activity defines colorectal cancer stem cells and modulates through AP1- and TCF-dependent mechanisms. Stem Cells..

[22] Zhang Z, Bu X, Chen H, Wang Q, Sha W (2016). Bmi-1 promotes the invasion and migration of colon cancer stem cells through the downregulation of E-cadherin. Int J Mol Med..

[23] Kimura Y (2013). CD44variant exon 9 plays an important role in colon cancer initiating cells. Oncotarget..

[24] Nakaya T (2014). KLF5 regulates the integrity and oncogenicity of intestinal stem cells. Cancer Res..

[25] Neumann J (2011). SOX2 expression correlates with lymph-node metastases and distant spread in right-sided colon cancer. BMC Cancer..

[26] Lobo NA, Shimono Y, Qian D, Clarke MF (2007). The biology of cancer stem cells. Annu Rev Cell Dev Biol..

[27] Clarke MF (2006). Cancer stem cells–perspectives on current status and future directions: AACR Workshop on cancer stem cells. Cancer Res..

[28] Moore, N. & Lyle, S. Quiescent, slow-cycling stem cell populations in cancer: a review of the evidence and discussion of significance. *J Oncol*. **2011** (2011). 10.1155/2011/396076 PMC2948913 20936110

[29] Pannuti A (2010). Targeting Notch to target cancer stem cells. Clin Cancer Res..

[30] Takahashi H (2011). Significance of Lgr5(+ve) cancer stem cells in the colon and rectum. Ann Surg Oncol..

[31] Gao T (2016). DCLK1 is up-regulated and associated with metastasis and prognosis in colorectal cancer. J Cancer Res Clin Oncol..

[32] Shimokawa M (2017). Visualization and targeting of LGR5+ human colon cancer stem cells. Nature..

[33] Morikawa K (1988). Influence of organ environment on the growth, selection, and metastasis of human colon carcinoma cells in nude mice. Cancer Res..

[34] Livak KJ, Schmittgen TD (2001). Analysis of relative gene expression data using real-time quantitative PCR and the 2(−Delta Delta C(T)) Method. Methods.

