# Supplementary material for: Sox2 is associated with cancer stem-like properties in colorectal cancer
Source: Sci Rep. 2018 Dec 5;8:17639. doi: 10.1038/s41598-018-36251-0 (PMC6281572; doi:10.1038/s41598-018-36251-0)
Supplement: Supplementary file 1 — Supplementary Figure [file 41598_2018_36251_MOESM1_ESM.pdf]

Sox2 is associated with cancer stem-like properties in colorectal cancer

Koki Takeda, Tsunekazu Mizushima, Yuhki Yokoyama, Haruka Hirose, Xin Wu, Yamin Qian, Katsuya Ikehata, Norikatsu Miyoshi, Hidekazu Takahashi, Naotsugu Haraguchi, Taishi Hata, Chu Matsuda, Yuichiro Doki, Masaki Mori, Hirofumi Yamamoto

Supplementary Table 1 Clinicopathological factors and Sox2 mRNA expression in 130 colorectal cancers

Patients Background	Low（N = 65)	High（N = 65)	*P* value
Gender			
Male / Female	34/31	36/29	0.725
Location			
Rectum / Colon	25/40	27/38	0.720
Histological type			
tub1 / tub2, muc, por	18/47	14/51	0.542
Depth			
T0, T1, T2, T3 / T4	32 / 33	31 / 34	1.000
Lymph node metastasis			
Negative / Positive	34 / 31	29 / 36	0.483
Distant metastasis			
Negative / Positive	55 / 10	51 / 14	0.498
Lymphatic duct invasion			
Negative / Positive	21 / 44	15 / 50	0.234
Venous invasion			
Negative / Positive	24 / 41	25 /40	0.856
pStage			
I, II / IIIa, IIIb, IV	31 / 34	24 / 41	0.287

Supplementary Table 2 Specific PCR primer pairs

Gene	Primer sequence (5' to 3')	
Sox2	F	ATCACCCACAGCAAATGACA
	R	GTGCAAAGCTCCTACCGTACCACTA
Oct-4	F	GACAACAATGAGAACCTTCAGGAGA
	R	CTGGCGCCGGTTACAGAACCA
Nanog	F	TTGTGGGCCTGAAGAAAACTATCC
	R	CTGCGTCACACCATTGCTATTCTT
Bmi-1	F	TGTAAAACGTGTATTGTTCGTTAC
	R	CAATATCTTGGAGAGTTTTATCTGACC
CD44v9	F	ATAATGAGCTTCTCTACATCACATGAAGGC
	R	ATAATGTCAGAGTAGAAGTTGTTGGATGGT
Klf-5	F	CCACCACCCTGCCAGTTAAC
	R	TAAACTTTTGTGCAACCAGGGTAA
Gapdh	F	CAACTACATGGTTTACATGTTC
	R	GCCAGTGGACTCCACGAC

A

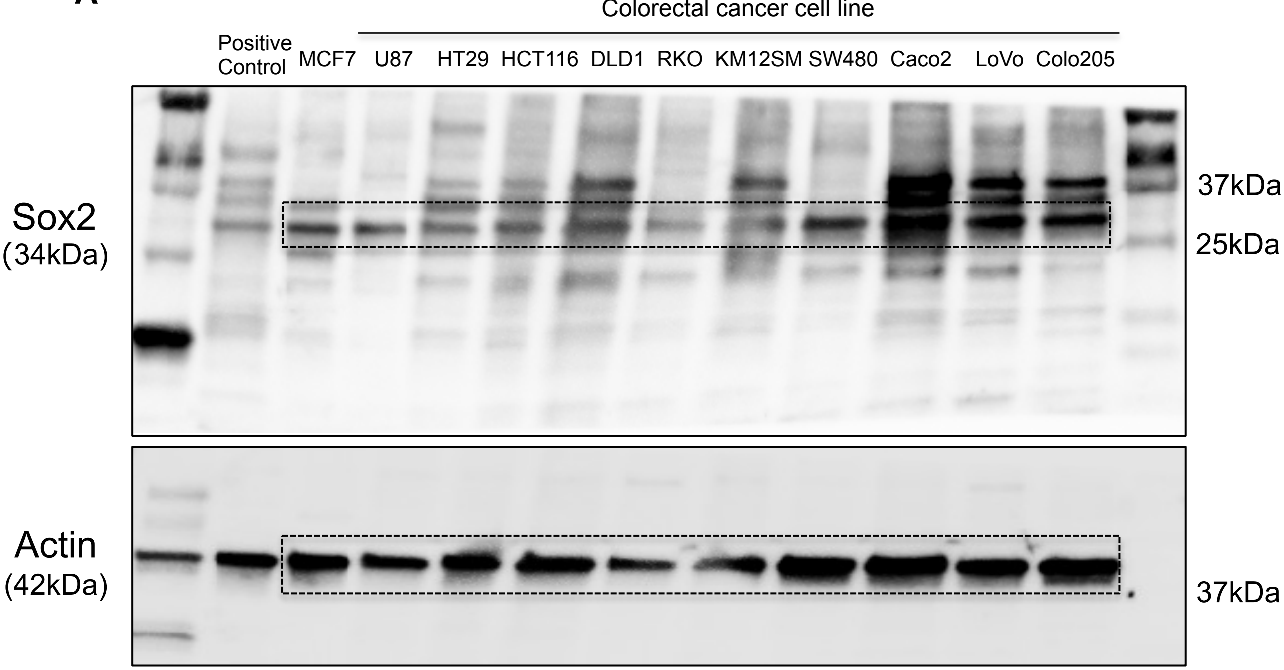

This area is “Figure1A”.

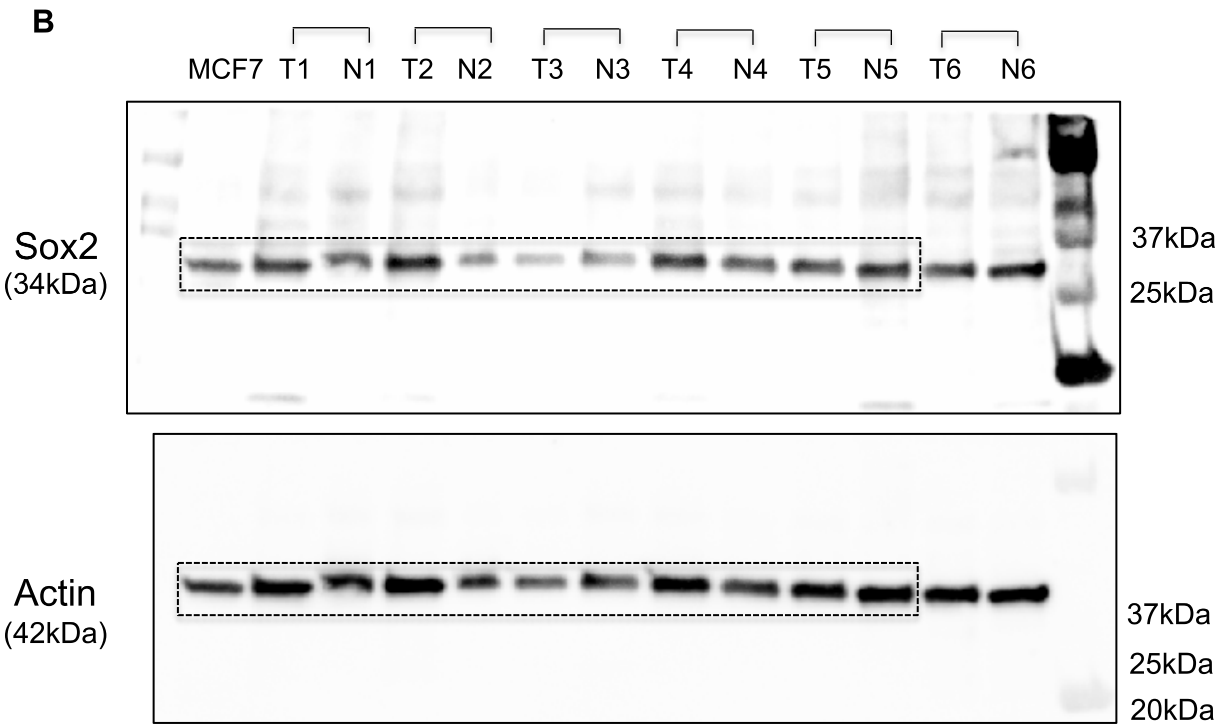

This area is “Figure1B”.

C

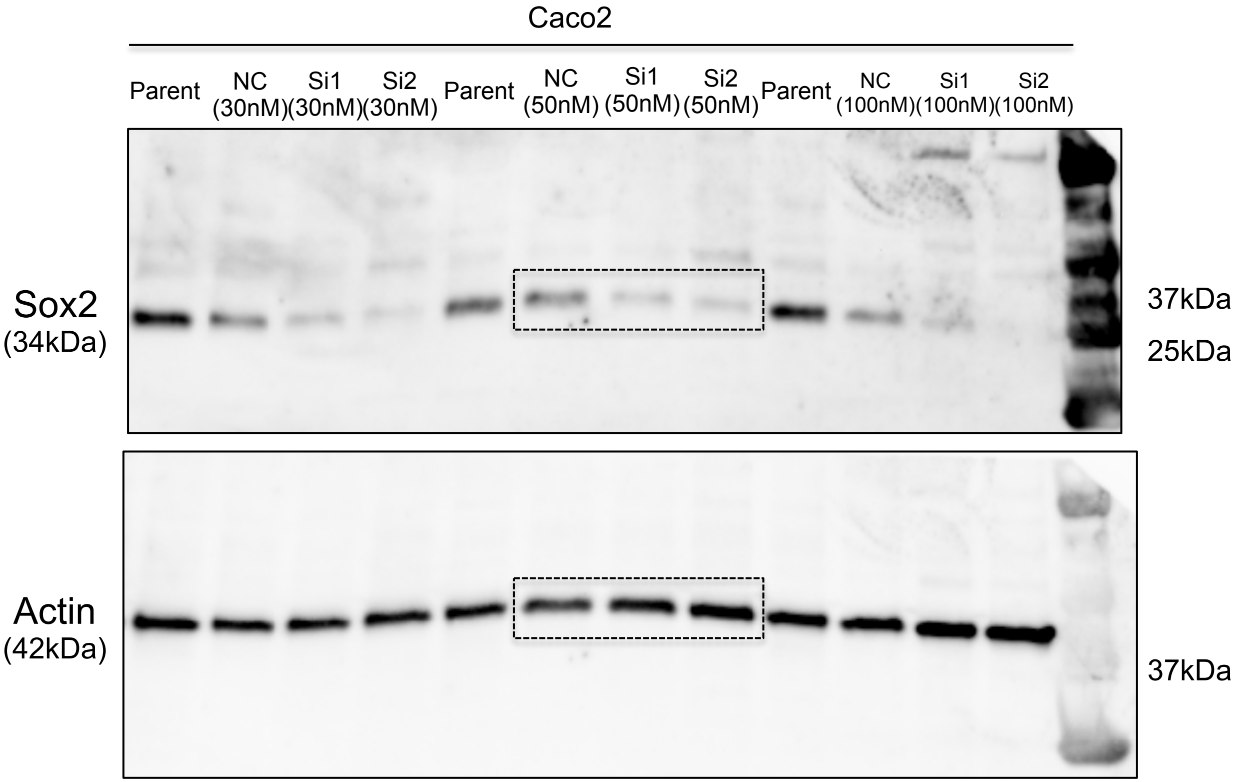

This area is “Figure1C”.
